# Multigene Copy Number Alteration Risk Score Biomarker–Based Enrichment Study Designs in Metastatic Castrate-Resistant Prostate Cancer

**DOI:** 10.1200/PO-24-00399

**Published:** 2024-12-03

**Authors:** Yeonjung Jo, Jonathan J. Chipman, Benjamin Haaland, Tom Greene, Manish Kohli

**Affiliations:** ^1^ Division of Biostatistics, Department of Population Health Sciences, School of Medicine, University of Utah, Salt Lake City, UT; ^2^ Cancer Biostatistics, Huntsman Cancer Institute, University of Utah, Salt Lake City, UT; ^3^ Pentara Corporation, Salt Lake City, UT; ^4^ Division of Oncology, Department of Medicine, Huntsman Cancer Institute, University of Utah, Salt Lake City, UT

## Abstract

**PURPOSE:**

A composite multigene risk score derived from tumor-biology alterations specific to metastatic castrate-resistant prostate cancer (mCRPC) state was evaluated as a classifier to design biomarker-based enrichment clinical trials.

**METHODS:**

A plasma cell-free DNA copy number alteration risk score based on alterations in 24 genes was simulated to develop a biomarker classifier–based clinical trial design enriched for high-risk patients to detect a survival advantage of a novel treatment (hazard ratio of 0.70 with 80% power). We determined the design trade-offs between the number of patients screened and enrolled when varying the type of patients to enrich and the extent of enrichment needed.

**RESULTS:**

For a 2-year overall survival end point in mCRPC state, fully enriching patients with mCRPC having a high-risk score of 3 or more (the 95th percentile of a range of risk scores in patients with mCRPC) was determined to require screening to a maximum of 4,149 patients to enroll 259 patients for the targeted effect size. A nonenriched trial was determined to require enrolling 689 patients to be equivalently powered. We identified a pragmatic alternative, which is to enrich patients with mCRPC with a risk score of 1 or more (the 67th percentile) and an enrichment fraction of 0.25. This would require screening 658 patients to enroll 584 patients, and it maximizes the ability to detect a difference in treatment effect by risk score.

**CONCLUSION:**

A plasma multi-CNA risk score classifier can feasibly be leveraged to design an enrichment trial in mCRPC. Enriching 25% of patients screened with a risk score >1 was observed to be optimal for obtaining an adequately powered, biomarker-based mCRPC-enriched clinical trial.

## INTRODUCTION

With an estimated 19.3 million cancer cases and an estimated mortality of nearly 10 million cancer-related deaths in 2021 worldwide,^1^ cancer remains a significant public health challenge. A decline in cancer mortality trends especially for lung cancer and melanoma among several other solid tumors in the United States and globally has been observed through 2021.^2^ Factors responsible for this may include the increase in early cancer screening and interventions for localized stage, reductions in smoking, access to treatments for nonlocalized stages, and the introduction of targeted molecular therapies. Since 2005, increase in US Food and Drug Administration drug approvals has occurred with more than 120 novel therapeutic drugs approved for the management of solid tumors alone.^3^ Modern cancer drug approvals have evolved from a focus on chemotherapeutic entities to small-molecule targeted drugs, monoclonal antibodies, antibody-drug conjugates, and immunotherapies.^3^ The publication of the human genome project and The Cancer Genome Atlas has in specific led to new molecular entities in cancer therapeutics that are based on subtyping and targeting the genetic heterogeneity of tumors, which is undecipherable with histology alone. Additionally, the molecular target-based approach of drug-biomarker pairing for prognosis and prediction of responses has introduced novel clinical study designs resulting in a paradigm shift that has introduced incorporation of biomarkers in clinical trial designs in recent years,^4^ which focus on the use of genomic signatures in randomizing treatments and enhancing novel therapies.^5,6^ Molecular prognostic biomarkers typically provide information about clinical outcomes regardless of the therapy based on expression patterns of a particular gene/pathway, whereas molecular predictive biomarkers provide information about the effectiveness of the treatment agent used.^7^ Since prognostic and predictive biomarkers are not mutually exclusive, a genomic signature initially developed can be a prognostic and/or predictive biomarker, but the purpose they are to be used for can affect designing biomarker-based clinical trials during the development process. In addition, genome-based molecular alterations that have been used in biomarker-based clinical trial designs have ranged from targeting single-gene alterations or a more expansive set of alterations using a composite algorithmic score for patient selection and screening before a score-based randomization of the interventions in prospectively conducted, predictive biomarker trials. Successful examples of single gene altered biomarker-based therapeutics include specific inhibitors to genomic aberrations in epidermal growth factor receptor exons (exon 19 deletion; exon 21 mutation or exon 20 T790M mutation), *ALK* fusions, *ROS-1* fusions, *RET* fusions, *MET* exon 14 splice mutation, and *KRASG12C* and *BRAFV600e* mutations which have led to significant improvements in survival in non–small-cell lung cancer.^8-15^ Similarly, successful targeting of *BRAF V600 E/K* mutation–positive metastatic melanoma has led to prolongation of survival.^16-18^ These biomarker-based clinical trial designs range from cohort studies to phase I to phase III and randomized to nonrandomized as well as open-label trial designs. Additionally, multiple stage-specific alterations used as composite risk scores have been developed as predictive biomarkers. The recurrence score of OnctypeDx in a 21-gene expression assay is an example that was prospectively tested in randomized clinical trials to decide on adjuvant chemotherapy use for breast cancer (RxPONDER, TAILORx).^19,20^ A more expansive 70-gene score panel (MINDACT)^21^ in early breast cancer is another example.

**CONTEXT**

**Key Objective**
We focused on developing an enrichment study design for treatment-naïve metastatic castrate-resistant prostate cancer (mCRPC) using a novel 24-multigene copy number alteration molecular score, leveraging the prognostic potential of this risk score to evaluate the overall benefit of additional treatments.
**Knowledge Generated**
Our simulations considered the trade-offs in clinical trials regarding the number of participants needed for screening and enrollment to demonstrate the effect of a novel therapy in reducing the hazard ratio of death compared with the standard of care. It was determined that enriching 25% of screened patients with a risk score >1 would be optimal for achieving a well-powered, biomarker-based mCRPC-enriched clinical trial. This approach would require screening 658 patients to enroll 584, sufficient to detect a difference in treatment effects based on the risk score.
**Relevance**
Development of innovative clinical trials that incorporate genome-based classifiers in future mCRPC clinical trial designs.

In advanced prostate cancer, therapeutic trial designs have targeted the homologous recombinant repair (HRR) pathway.^22-26^ These trials do not apply to the vast majority of patients with metastatic castrate-resistant prostate cancer (mCRPC) without HRR alterations. Biomarker trial designs on the basis of a composite multigene risk score reflective of tumor biology in mCRPC state is lacking in clinical trial designs,^27^ either for prognostication or for predictiveness of a new drug or drug combination. We identified a multigene copy number alteration (CNA)–based molecular score in mCRPC that has been demonstrated in multiple mCRPC cohorts to have prognostic value and also associate with therapy response (to abiraterone acetate/prednisone).^28^ We were interested to develop a class of enrichment study designs in treatment-naïve mCRPC state using this novel multigene CNA molecular score drawing upon the reported prognostic nature of the biomarker risk score to test the overall benefit of additional novel treatments. Secondary to the overall benefit, we also explored the ability to detect whether the novel treatment has a predictive treatment effect using a genomic risk score classifier in a biomarker-based clinical trial design approach.

## METHODS

### Multigene, CNA Classifier Description

A composite 24-gene–based classifier of clinical outcomes in mCRPC previously reported from a prospective biospecimen collection (N = 87) cohort study (ClinicalTrials.gov identifier: NCT01953640)^28-30^ and in a real-world data repository (N = 92) was evaluated for designing a biomarker-based clinical trial in mCRPC. We used the 24-gene classifier generated and reported using a prospective cohort study of 87 patients with mCRPC enrolled between May 2013 and December 2018 in whom biomarker defining guidelines for survival and therapy response to abiraterone acetate/prednisone (AA/P) was measured as per the Prostate Cancer Working Group-2 (PCWG2)^31^ criteria guidelines to perform a simulation study of designing enrichment-type biomarker-based clinical trials.^28-30^ The biomarker classifier is a composite risk score that is determined on the basis of CNAs observed in 24 genes in cell-free DNA (cfDNA) and which was associated with overall survival (OS) and therapy response to AA/P.^28^ The composite risk score was developed by estimating the gain or loss of the CNA status for each of the 24 genes and the hazard ratio (HR) for survival relative to no change in CNA status. A positive coefficient was associated with worse OS. The composite risk scores were then calculated for OS and for progression-free survival (PFS) using the following formula:

Sum [Cox regression coefficient×CNA status (1 for gain or loss,and 0 for no change) of each gene selected]

The 24 genes include *AR*, *PTEN*, *RB1*, *TMPRSS2*, *MYCL*, *MYC*, *NOTCH1*, *TP53*, *ERG*, *FOXA1*, *NKX3.1*, *ZBTB16*, *NCOR1*, *NCOR2*, *COL22A1*, *PIK3CA*, *PIK3CB*, *LIK3R1*, *BRAF*, *RAF1*, *SPOP*, *APOB*, *CHD1*, and *CCND1*. A risk score was determined for each patient with mCRPC for both OS and PFS to AA/P as well as a range of risk scores in the cohort. This range was dichotomized at the median for both clinical end points of OS and therapy response to AA/P (PFS), with patients with mCRPC having scores above this median classified as high-risk as they were observed to have significantly shorter OS and therapy response (PFS) to AA/P. Data Supplement, Figure S1 shows the regenerated composite risk score on the basis of 24 candidate genes using the same method for the 87 patients in the cohort study^28^ for OS as a histogram, and Kaplan-Meier curves for OS by the multigene risk scores are provided in Data Supplement, Figure S2 for all patients and subgroups of patients with increasingly high multigene risk scores. The risk score models were internally validated using leave-one-out cross-validation.

### Enrichment Study Design Based on the Multigene CNA Risk Score for Enhancing Survival

**FIG 1.**
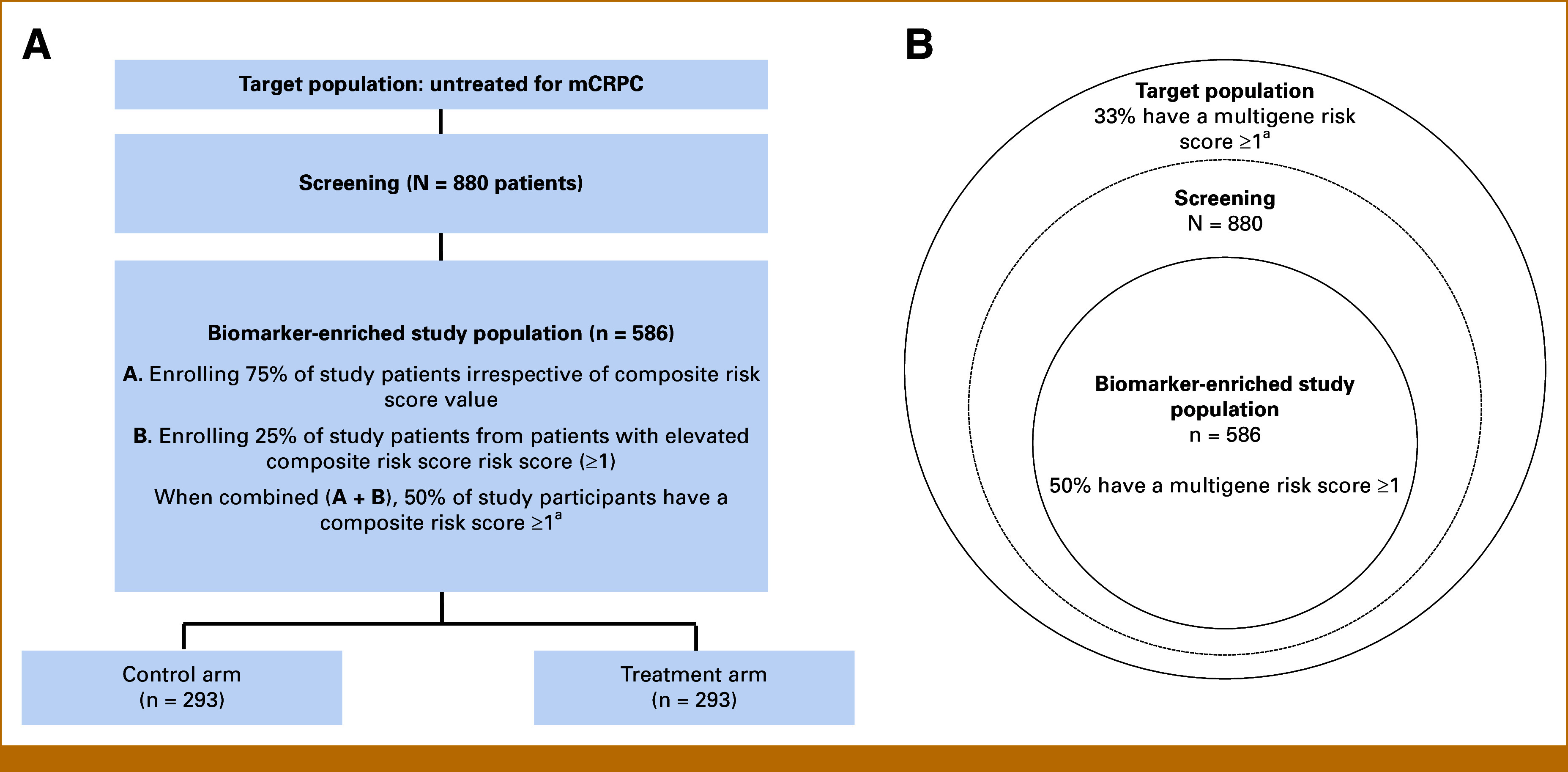
Visualization of the study design; (A) when the study enrichment fraction is 25% (η=0.25), threshold is 1 (δ=1), and follow-up time is 2 years. (B) Enriching on these patients creates a study population with half of the patients having a composite risk score ≥1. ^a^Based on 33% of PROMOTE Cohort having a composite risk score ≥1. mCRPC, metastatic castrate-resistant prostate cancer.

For concreteness, we considered the challenge of designing a trial that is enriched by oversampling high-risk patients to reduce the required number of randomly designed patients for a biomarker-based enrichment trial in patients with mCRPC with aggressive tumor biology defined by a multigene CNA risk score. As a frame of reference, the biomarker-based design we consider targets enrolling patients with mCRPC to be 80% powered to detect improved OS, as measured by an HR of 0.70, for all patients randomly assigned to a novel (future) treatment compared with the standard of care while maintaining a 5% type I error rate. Although patients with a higher composite risk score are more likely to experience an event, we initially designed the trial as though there is a constant treatment effect regardless of the risk score value. A trial with equal allocation to treatment and control arms requires observing 247 events to be sufficiently powered; hence, enriching patients with high survival outcomes could reduce the overall required sample size. A secondary goal of the enrichment trial would be to test for the presence of treatment effect heterogeneity across different levels of the mutigene score (ie, predictability). Using data from the cohort study,^28^ we estimated through simulations the attributes of various enrichment trial study designs that would be adequately powered for the primary analysis. We considered the effect of determining the patient type to enrich on the basis of CNA score (called *enrichment threshold*) and the extent to which the study will enrich these patients (called *enrichment fraction*). Enriching high-risk patients increases the expected number of observed outcomes. Although this may increase the number of patients to be screened, it reduces the total sample size and can increase the ability to detect the predictiveness of the CNA risk score. We chose a study follow-up for *t* = 24 months on the basis of expected survival rates of patients with mCRPC from published reports^32,33^ and also considered study follow-up for *t* = 36 months. The median survival of treatment-naïve patients with mCRPC reported in most recent randomized clinical trials using androgen receptor pathway inhibitors is between 30 months and 34 months.^32-34^ A graphical illustration of the study design is provided in Figures 1A and 1B.

The number of patients to screen for the trial depends on the enrichment threshold (δ), enrichment fraction (η), and the required sample size (n) to achieve the desired number of events:

$$s=\eta \frac{n}{prevalence\;above\;\delta }+\left(1-\eta \right)n$$

We used Cox proportional hazards model for sample size and power calculations, but the proportional hazard model assumption would need to be evaluated for actual studies. Details on estimating the expected number of events and required sample size for the enriched population are provided in the Data Supplement. Further details are also provided for power calculations to detect a treatment effect by patients above versus below the enrichment threshold.

For exploration, we considered varying the design parameter enrichment threshold (δ) from –3 (1st percentile) to 3 (95th percentile) for the CNA score and in increments of 1, and we considered varying enrichment fraction (η) from 0 (no enrichment) to 1 (full enrichment) by increments of 0.25. Further details for each enrichment threshold and fraction combination are provided in the Data Supplement. The operating characteristics of alternative designs were evaluated using statistical simulation on the basis of inputs that were estimated from observed data. We used bootstrap CIs, drawn from 1,000 bootstrap samples, to quantify the uncertainty in the operating characteristics resulting from sampling error in the inputs that were estimated from the data.

## RESULTS

**FIG 2.**
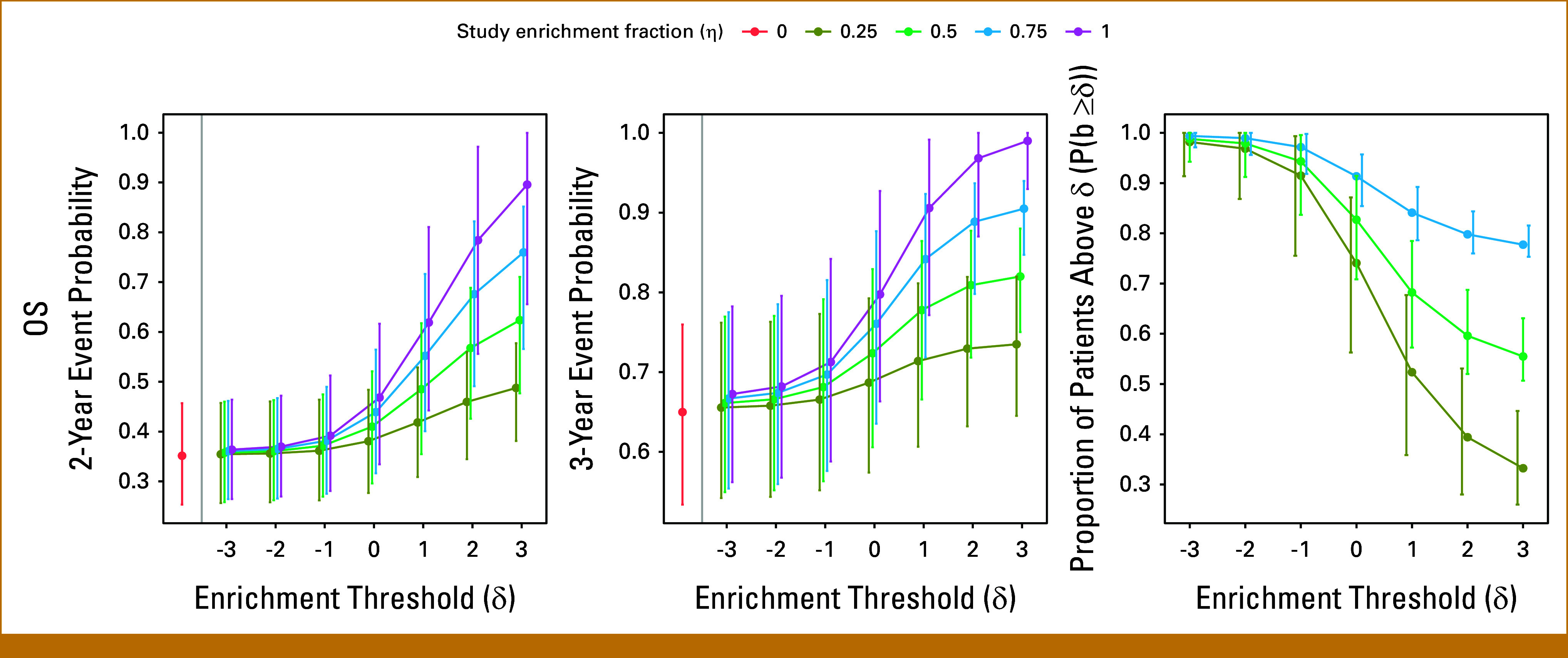
Estimated event probability and proportion of patients above the enrichment threshold in a study population and 95% bootstrap CIs by design enrichment threshold and fraction. OS, overall survival.

**FIG 3.**
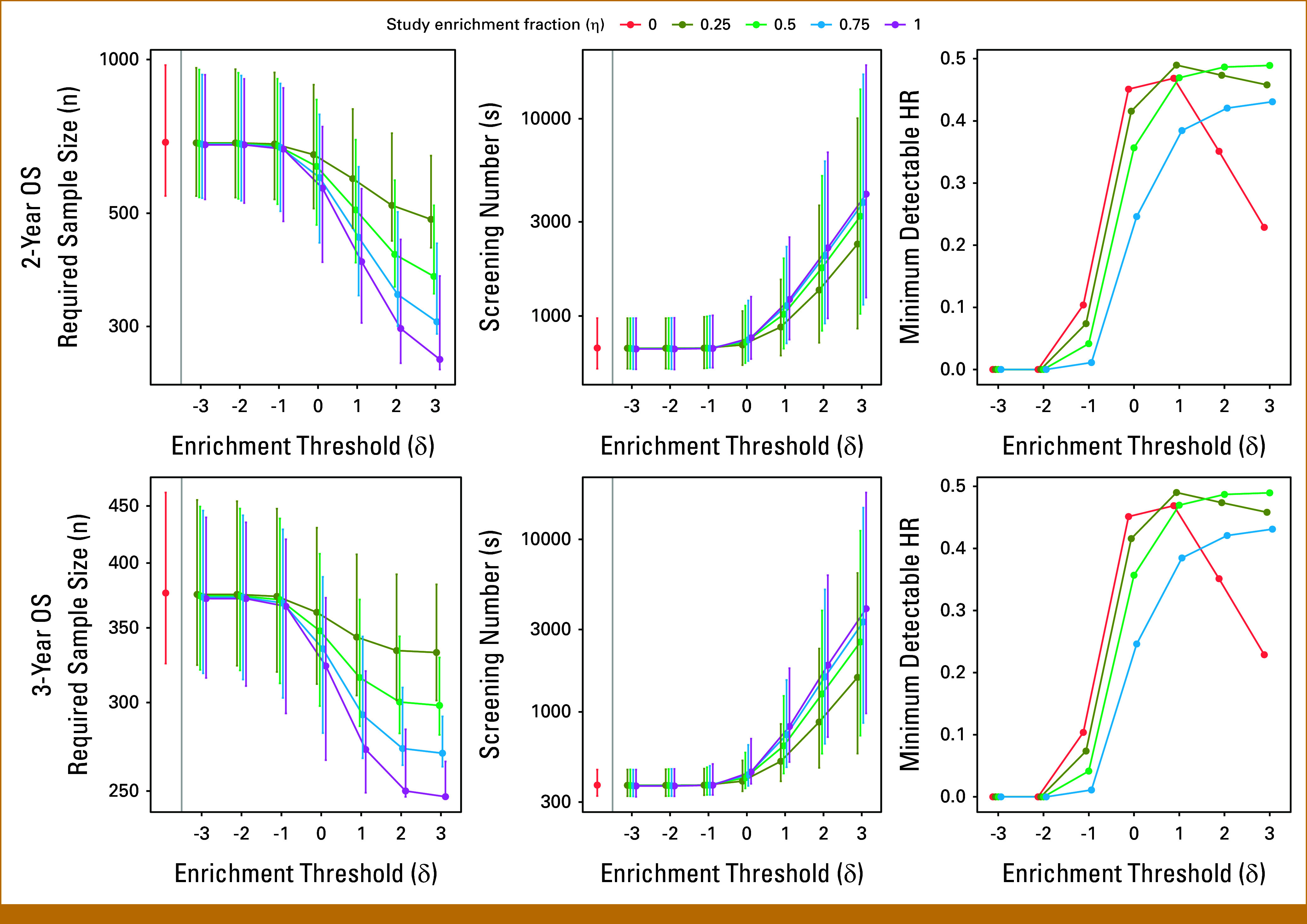
Estimates and 95% bootstrap CIs of the required sample size to detect an overall HR of 0.70 with 80% power, the number of patients needed to screen, and the minimum detectable HR to achieve 80% power to test the predictability of a treatment among those above versus below the enrichment threshold. For the interaction test, a minimum detectable HR close to 0 corresponds to a very large effect, and a small effect is an HR close to 1. HR, hazard ratio; OS, overall survival.

Data Supplement, Figures S1 and S2 provide the plasma cfDNA multigene risk score classifier ranges for prognostication of OS in mCRPC state and the OS for each composite risk score. The estimated event probabilities of the target population (η=0) are 0.36 for 2-year OS and 0.66 for 3-year OS. As the study enrichment fraction increases, meaning that more patients with a high risk of death are included, the study event probability increases. Although this can decrease the overall sample size required for adequate power, it can increase the screening number required to obtain this sample size. The trade-offs between these design attributes are driven by the selected study enrichment threshold and the enrichment fraction. The estimated event probability and the proportion of the study having a multigene risk score greater than the enrichment threshold (δ) in each design are provided in Figure 2, and the effect on the required sample size and required number screened is provided in Figure 3 for 2- and 3-year study follow-up.

As the enrichment threshold increases, the required sample size decreases, and the screening number generally increases. The enrichment trial design that required the smallest sample size was to fully enrich patients (η=1) with a multigene risk score of 3 in all scenarios; however, this design required the greatest number of patients to be screened. Considering 2-year study follow-up for an OS end point, with enrichment fractions of 0.25, 0.5, 0.75, and 1, a total of 2,316, 3,205, 3,764 and 4,149 patients would need to be screened to fulfill the required sample size of 487, 376, 307, and 259 to ensure 80% power when the enrichment threshold is 3. For a 3-year follow-up on an OS end point, with enrichment fractions of 0.25, 0.5, 0.75, and 1, a total of 1,584, 2542, 3,320, and 3,965 patients need to be screened to fulfill the required sample size of 333, 299, 271, and 248 to ensure 80% power when the enrichment threshold is 3 for the CNA score.

The number of patients needed to be screened generally increases when setting a higher enrichment threshold and enrichment fraction. For a 2-year and 3-year OS end point, the screening number is essentially the same for all enrichment fractions when the enrichment threshold is <0. The screening number slightly decreases when the outcome is 2-year OS, the enrichment threshold is 0 or 1, and the enrichment fraction is 0.25. And, the screening number slightly decreases when the outcome is 3-year OS, the enrichment threshold is 0, and the enrichment fraction is 0.25.

Given the sample sizes to detect an overall effect, we also compared the minimum detectable treatment effect modification by being above versus below the multigene risk score threshold to evaluate the CNA score as a therapy predictive classifier. The trial designs best suited to detect a differential effect are ones that, first, enrich upon patients who have a multigene risk score of 1 or more with an enrichment fraction of 0.25 and, second, enrich upon patients who have a multigene risk score of 2 or more (or 3 or more) with an enrichment fraction of 0.5 (Fig 3). Although the detectable effect modification under 80% power is a relatively large HR of 0.49, this is the smallest detectable effect modification across designs. These designs are optimal for assessment effect modification because roughly half of the patients enrolled would be above versus below the enrichment threshold. Estimates corresponding to Figure 3 are provided in Data Supplement, Table S1.

## DISCUSSION

When there is initial evidence that a biomarker is prognostic of clinical outcomes and possibly predictive of treatment effects, enrichment designs can leverage this information for a more efficient clinical trial. In our simulations, we evaluated a tumor-biology–based plasma cfDNA multigene CNA risk score as a classifier determined in cohort studies^35^ to have prognostic value for OS in mCRPC state, for being tested in prospective enrichment-style biomarker-driven clinical designs. Of note, in mCRPC state, molecular prognostic determinants are lacking but provide the advantage of being targetable therapeutically, unlike nonspecific clinical laboratory prognostic measures in this stage including hemoglobin, lactate dehydrogenase, and alkaline phosphatase levels.^36^

We observed that screening up to a maximum of 4,149 patients in mCRPC state and enrolling up to 689 patients are feasible to conduct in a multicentered clinical trial, and thus an enrichment trial design best suited to detect a difference in treatment effect above versus below an enrichment threshold is achievable to perform (Fig 3). In our simulation study, we determined that designs that are the combinations of the study enrichment fraction of 0.25 with an enrichment threshold of 1 and the study enrichment fraction of 0.5 with an enrichment threshold of 2 are practical. It is difficult to determine or prespecify a constant enrichment threshold for all studies. For each scenario of enrichment fraction and threshold considered, study logistics play a key role in determining the optimal enrichment trial design that is plausible to conduct. As an example, Figure 1 provides a scenario for using a biology-based classifier to design a clinical trial on the basis of biomarker classifier–driven risk scores with optimal thresholds and enrichment fraction to conduct a biomarker classifier–driven trial in patients with mCRPC. When the availability of patients is limited, the number of patients available for screening becomes the first limitation. The costs of screening, patient treatment, and follow-up become the next considerations. If an ample number of patients can be screened at an affordable cost of screen and patient follow-up, the trial can prioritize powering the study to test the predictive nature of multigene risk score. With an enrichment trial, the attempt is to aim to have continual enrollment to both the enriched and nonenriched study participants to avoid confounding by time trends. In addition, one can also incorporate a prespecified retention rate after screening to estimate a precise screening number.

The paradigm for probing genomic classifiers initially from the prognostic risk category that defines high risks to predictive ones has precedence in clinical trial designs with the Recurrence Score in breast cancer.^20,37^ The 21-gene expression score was initially used as a prognostic factor in 2006 and thereafter was able to define in the TAILORx trial which patients needed any adjuvant therapy on the basis of the score calculus—in other words, the potential use as a predictive biomarker. As such, we consider the score at present as attempting to define subcohorts of patients with lethal mCRPC on the basis of genomic alterations and not a mechanistic tool ready for use to decide on specific treatments.

However, there are limitations to our study that also include calculation of multigene risk scores in the general population. The Cox regression coefficients used to calculate the biomarker-based score in our previous study was validated predominantly in White populations and did not include patients of African American, Hispanic, and Asian descents. The predictability of the multigene risk score was validated using leave-one-out cross-validation as well.^35^ Developing a standardized and universal multigene risk score for broad application in mCRPC is therefore needed. Additionally, the study design focuses on prognostic value for the classifier and the treatment and classifier interaction and may not provide guidance for choosing specific therapies that are based on the classifiers when the treatment-classifier interaction is not sufficiently powered for choosing a specific therapy.

In conclusion, we introduce a simulation of using a plasma multi-CNA risk score classifier that can feasibly be leveraged to design an enrichment trial in patients with mCRPC, and while establishing the plausibility of the risk score to be used as a classifier, refinement of the risk score for designing an optimal enrichment trial is achievable to further mCRPC state therapeutics that are based on tumor-biology in future.
